# Dynamic Performance of a Steel Road Sign with Multi-Material Electronic Signboard Under Mining-Induced Tremors from Different Mining Areas: Experimental and Numerical Research

**DOI:** 10.3390/ma18071451

**Published:** 2025-03-25

**Authors:** Paweł Boroń, Joanna Maria Dulińska

**Affiliations:** Faculty of Civil Engineering, Cracow University of Technology, 31-155 Cracow, Poland; joanna.dulinska@pk.edu.pl

**Keywords:** multi-material electronic signboard, steel road sign, mining-induced tremor, operational modal analysis, numerical simulation, numerical model validation, experimental verification, dynamic response to mining excitation

## Abstract

This study investigates the dynamic performance of a road sign equipped with a multi-material electronic signboard subjected to mining-induced seismic tremors. The key innovative aspect lies in providing new insights into the dynamic performance of multi-material electronic signboards under high-energy mining tremors, enhancing their safety assessment in mining areas. Experimental modal analysis and finite element analysis were conducted, and the numerical model of the sign was calibrated by adjusting ground stiffness to align experimental and computational data. The fundamental natural frequencies and their corresponding mode shapes were identified as 2.75 Hz, 3.09 Hz, 8.46 Hz, and 13.50 Hz. Numerical results were validated using MAC methods, demonstrating strong agreement with experimental values and confirming the accuracy of the numerical predictions. Damping ratios of 3.79% and 3.71% for the first and second modes, respectively, were measured via hammer tests. To evaluate the sign’s dynamic performance under high-energy mining-induced tremors, two events were applied as kinematic excitation of the structure. These tremors, recorded in different mining regions, exhibited significant variations in peak ground acceleration (PGA) and dominant frequency range. A key finding was that frequency matching between the dominant frequencies of the tremor and the natural frequencies of the sign had a greater impact on the sign’s dynamic response than PGA. The Szombierki tremor, with dominant frequencies of 1.6–4.8 Hz, induced significantly higher stress and displacement compared to the Moskorzyn tremor (5–10 Hz) despite the latter having twice the PGA. These results highlight that a road sign structure can exhibit widely varying dynamic behaviors depending on the seismic characteristics of the mining zone. Therefore, a comprehensive assessment of mining-induced tremors in relation to the seismicity of specific areas is crucial for understanding their potential impact on such structures. The dynamic performance assessment also revealed that the electronic multi-material signboard did not undergo plastic deformation, confirming it as a safe material solution for use in mining areas.

## 1. Introduction

The negative impacts of mineral extraction and mining activity on land quality [1,2] must be balanced with the implementation of effective measures to protect the environment and civil infrastructure [3]. The extraction of coal, oil, and copper plays a significant role in generating both static (resulting from ground deformation) [4,5] and dynamic (originated from mining-induced seismicity) loads, which greatly impact engineering structures [6,7]. Particularly, vibrations from mining-induced tremors can lead to structural damage, compromising the safety and stability of buildings and roads.

In recent years, increasing attention has been given to the risks associated with mining-induced seismic activity, as the energy release and vibration amplitudes of high-intensity mining tremors often reach levels comparable to minor earthquakes. However, mining-induced tremors differ from natural seismic events in several aspects.

Mining-induced events are generally weaker but more frequent than earthquakes. While earthquakes can last minutes, mining tremors typically last up to 10 s, with the most intense phase lasting 0.5–5 s [4,8]. These events have higher dominant frequencies (2–7 Hz) than natural seismic events (0.5–2 Hz) [9,10]. Their characteristics, including peak ground acceleration and frequency range, vary by mining zone due to geological conditions [11]. Earthquakes produce stronger horizontal vibrations, whereas mining-induced events have similar amplitudes in all directions, sometimes with greater vertical movement [12].

Most studies on the impact of mining-induced shocks on surface structures have primarily focused on residential buildings (e.g., [13,14,15]), while the effects on other engineering structures remain insufficiently explored. For instance, mining-induced seismicity can significantly impact roads and auxiliary infrastructure, such as road signs. Strong vibrations may loosen or misalign signposts, causing them to shift or even collapse, which can reduce the visibility of critical traffic information and increase the risk of accidents. Prolonged exposure to tremors can also weaken the structural integrity of sign supports, making them more vulnerable to damage from strong winds or vehicle collisions. Electronic traffic boards and signaling systems are also at risk, as vibrations may disrupt their operation, leading to malfunctions or inaccurate displays.

The dynamic performance of road signs has been extensively studied in the literature, with most research focusing on wind effects [16,17,18] or operational impacts like road traffic [19,20]. However, very few studies consider the effects associated with the response of auxiliary road infrastructure to ground motion excitations. When addressed, it concerns the dynamic response of such structures to natural seismic shocks [21,22,23,24,25].

The significant impact of seismic excitations on support structures was demonstrated in [21], which analyzed a steel truss portal used for road traffic signaling. That study revealed that ground motion induced high stress, particularly at column–beam joints, potentially causing severe damage or structural failure. Similarly, article [22] examined the seismic response of traffic light poles with three different mast arm lengths, evaluating their behavior under six recorded and two synthetic earthquake events. The correlation between seismic-induced forces and the stability and longevity of structures such as road signs and traffic signal poles was also demonstrated in [23]. This study showed that due to the close proximity of the natural vibration frequencies of the light poles and the bridge, resonance phenomena occurred, leading to the bending failure of the structure. Additionally, the exceedance of material strength limits in road infrastructure elements due to seismic excitation was documented in [24]. Identifying excessive stress levels in structural components provided a foundation for assessing the potential application of vibration-damping mechanisms in such structures. The study presented in [25] investigated the seismic response of cantilevered sign support structures used in highway systems. The findings highlighted the necessity of implementing vibration dampers to reduce both displacement and acceleration of the sign structure, ultimately enhancing its service life.

In summary, seismic-induced excitation can play a crucial role in the design of road signs. However, a significant gap remains in recognizing how mining-related shocks affect these structures. This lack of comprehensive studies has motivated the authors to explore the impact of mining-induced tremors on these types of engineering structures.

Although road signs are standardized prefabricated structures, their dynamic response levels may vary significantly depending on the specific mining area in which they are located. This variation is primarily due to differences in seismic activity characteristics, such as peak ground acceleration, dominant frequency range, and ground vibration patterns, which are unique to each region. Even though the structural design of road signs remains the same, the interaction between their natural frequencies and the local seismic excitation can lead to vastly different dynamic behaviors. This highlights the importance of considering local seismic conditions when assessing the dynamic performance of road signs installed in mining-affected areas.

A comprehensive assessment of mining-induced tremors in relation to the seismicity of specific areas is essential for identifying their potential impact on such structures. Predicted maximum PGA values in certain regions suggest a significant seismic impact, highlighting the need for continuous monitoring and evaluation to ensure the structural safety of road infrastructure, particularly in areas prone to increased mining-induced seismicity.

The primary objective of this study is to assess the dynamic performance of a typical road sign subjected to mining-induced seismic shocks. Specifically, this study aims to achieve the following:

1. Investigate how variations in the dominant frequency range of different mining activity areas influence the sign’s dynamic behavior;
2. Conduct a preliminary assessment of whether standard prefabricated road signs, particularly those with multi-material electronic signboards, provide a safe and effective material solution in mining areas.

The novelty of this study lies in its comprehensive evaluation of the dynamic response of a road sign equipped with a multi-material electronic signboard to mining-induced shocks. The key innovative aspects include the following:

- Comparing the dynamic performance of a road sign in two distinct mining regions, highlighting how regional variations in seismic excitation characteristics affect the dynamic response of a steel road sign;
- Utilizing an experimentally validated numerical model of a road sign to improve the accuracy of vibration predictions;
- Providing new insights into the dynamic performance of multi-material electronic signboards under mining tremors, enhancing their safety assessment in mining areas.

To the best of the authors’ knowledge, these aspects have not been addressed in the existing literature, making this study innovative.

The structure of this research is as follows:

▪ Presentation of the geometric and material details of a typical road sign structure, the description of its numerical model, and theoretical background along with the experimental set-up for estimating modal properties (Section 2);
▪ Experimental and numerical modal analysis to determine the natural frequencies and mode shapes of the typical road sign structure (Section 3.1 and Section 3.2);
▪ Verification and calibration of the numerical model of the sign (Section 3.3);
▪ Evaluation of the dynamic response of the road sign subjected to seismic shocks from two different mining regions (Section 4.1);
▪ Prediction of the potential plastic behavior of the multi-material electronic signboard under mining-induced tremors with anticipated maximum PGA (Section 4.2);
▪ Conclusions (Section 5).

## 2. Materials and Methods

### 2.1. Structural Morphology of the Analysed Steel Road Sign

**Figure 1:**
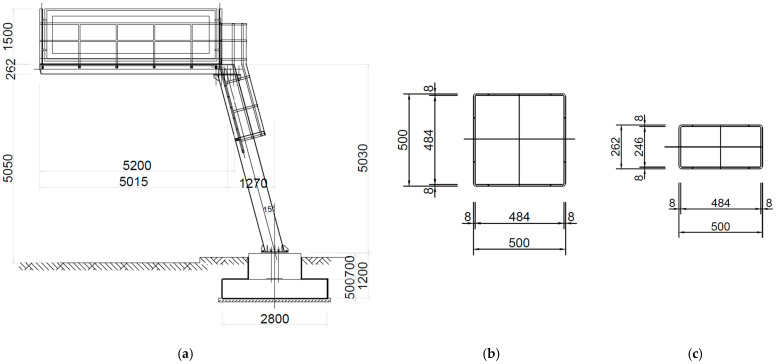
The analyzed steel road sign: (**a**) the main dimensions of the structure, (**b**) the cross-section of the steel support tower, (**c**) the cross-section of the cantilever arm.

The analyzed road sign is part of the infrastructure of an expressway located in Kraków, Poland. Its morphology is based on typical structural components, such as a concrete foundation, a steel load-bearing structure (an inclined support tower and a cantilever arm), and an electronic signboard ERGB 18.75-48 × 48 + ERGB 25-120 × 40. Additionally, the cantilever arm is equipped with a gusset plate and railings that enable safe maintenance work. These elements also provide additional reinforcement and rigidity to the entire structure. The basic dimensions of the road sign and cross-sections of the inclined column and cantilever arm are presented in Figure 1.

The concrete foundation block has dimensions of 2800 × 2000 mm and is embedded 1200 mm into the ground. The total height of the load-bearing structure is 5030 mm, while the length of the cantilever arm is 5200 mm. The inclined column supporting the signboard has a box cross-section with dimensions of 500 × 500 mm, and the thickness of the steel wall is 8 mm. The cantilever arm has a cross-section of 500 × 262 mm, with wall thickness also being 8 mm. The support structure is anchored to the concrete foundation using bolts of type M20 class 5.6.

The 1400 × 4600 mm electronic signboard is made from multi-material components (LCD panel/steel/aluminum/plastic). It is attached to the supporting structure using special bolts equipped with a sign. Electronic signboards are made from a combination of materials designed to ensure durability, visibility, and resistance to environmental conditions. The display panel consists of LED modules, typically covered with a polycarbonate or tempered glass protective layer to enhance visibility and shield against damage. The electronic components, such as circuit boards, are usually made from epoxy–fiberglass composite, ensuring good electrical insulation and mechanical stability. These materials together create a robust and long-lasting system capable of operating in harsh outdoor conditions. For the preliminary investigation into the possibility of plastic deterioration or any damage to the signboard under mining-induced shocks, the elastic–plastic homogeneous material for the entire signboard was used based on the literature data [26,27,28].

Table 1 summarizes the basic material data for the individual components of the road sign structure.

### 2.2. In-Field Tests and Experimental Set-Up

**Figure 2:**
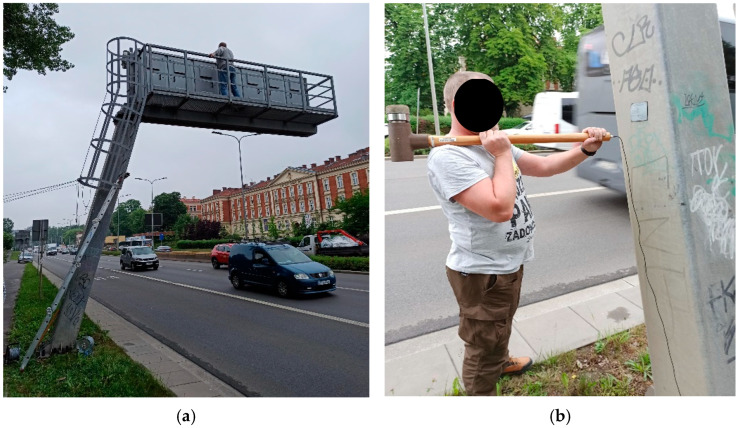
Types of excitations: (**a**) vehicle movements in road traffic, (**b**) modal hammer impacts.

The modal parameters of the road sign were experimentally determined through the measurement of vibrations induced by two types of excitations. Firstly, to identify natural frequencies and modes of vibrations of the structure, measurements were carried out using vibrations caused by various types of vehicle movement in road traffic (Figure 2a). Due to the anticipated influence of the type and level of excitation on the dynamic response of the road sign, two different types of traffic excitation were considered during the experiment planning: light traffic (primarily passenger cars) and heavy traffic (daily traffic). It was assumed that the dynamic characteristics of the structure obtained based on varying excitation levels would be more reliable. Secondly, to recognize the damping properties of the structure, modal hammer excitations generated vibrations of the road sign (Figure 2b). Table 2 introduces the labels of the measurements used in the subsequent sections of this article, along with the description of each test.

**Figure 3:**
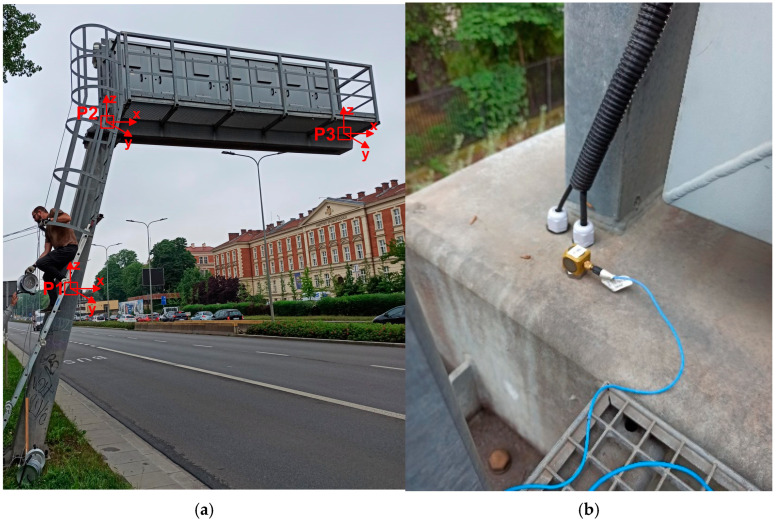
Experimental set-up: (**a**) the configuration of the measurement points and the directions of vibration registration, (**b**) the exemplary accelerometer located at point P2.

To acquire vibrational data, three measurement points (P1, P2, and P3) were established. The configuration of the measurement points, as well as the directions of vibrations, are shown in Figure 3a. Point P1 was located at the mid-height of the inclined tower; point P2 was placed at the junction between the tower and the cantilever arm (Figure 3b), and point P3 was situated at the end of the arm. Each point was fitted with a high-sensitivity piezoelectric accelerometer (356B18 manufactured by PCB Piezotronics Inc.), capable of detecting vibrations in three directions. The accelerometers had a frequency range from 0.5 to 3000 Hz, a sensitivity of 1000 mV/g, and a measured range of +/− 5 g. All sensors were connected via wires, with the signal being sampled at a rate of 1024 Hz.

### 2.3. Theoretical Background

In the experimental part of this study, ambient vibrations caused by traffic and wind were taken into consideration, and Operational Modal Analysis (OMA) techniques were employed [29,30]. The OMA techniques used in this paper were Stochastic Subspace Identification (SSI) and Time Domain Decomposition (TDD) methods, which rely on signals registered in the time domain [31,32] (see Table 2). The key advantage of this method is that no signal filtering is required [33]. The SSI algorithm uses Complex Mode Indicator Functions (CMIF) to determine the natural frequencies of a structure [34,35]. Peaks in the CMIF plot indicate modes, and their corresponding frequencies provide the natural frequencies for each mode. The accuracy of the modal properties depends on selecting the appropriate modal parameters, such as model order (the system’s number of degrees of freedom) and convergence criteria, including frequency error, damping error, and modal vector similarity [36,37]. The value of the model order should be derived from the number of experimental sets and correspond to the required number of modes. The modal vector similarity specifies the minimum required degree of similarity of eigenvectors [36]. Based on the experimental set-up, the following parameters for the CMIF method were used in this study: model order 20; percent errors for stability criteria 5% for frequency and damping error; and 90% modal convergence criterion.

For each natural frequency of the road sign, the corresponding mode shape was identified based on the TDD method [30,38]. The result of the signal analysis using TDD is the amplitude of the mode shapes defined at individual measurement points.

In the process of verifying the mode shapes obtained from both experimental and numerical modal models of the road sign, the mathematical method known as the Modal Assurance Criterion (MAC) was employed in this work [39,40] (see Table 2). The MAC values for the *i* and *j* eigenvectors were calculated using the following formula:

(1) $$MAC(i,j)=\frac{{\left({\left\{\Psi_{i}\right\}}^{T}\left\{\Psi_{j}\right\}\right)}^{2}}{\left({\left\{\Psi_{i}\right\}}^{T}\left\{\Psi_{i}\right\})({\left\{\Psi_{j}\right\}}^{T}\left\{\Psi_{j}\right\}\right)},$$

where $\Psi_{i}$, $\Psi_{j\;}$ represent the modal vectors.

A *MAC*(*I*,*j*) value of 1 indicates identical mode shapes, while a value of 0 indicates entirely different eigenvectors. In practice, *MAC*(*I*,*j*) values between 0.8 and 0.2 are considered indicative of a valid modal model [40]. In this study, three stages of using the MAC method can be identified. Initially, the experimentally obtained mode shape vectors were used to assess the adequacy of the measurement point placement. During this phase, the experimental mode shape vectors were compared with each other, a process known as AutoMAC [39,40]. Next, *MAC*(*I*,*j*) was applied to compare the modal models resulting from different experiments. Finally, *MAC*(*I*,*j*) was used to validate the numerical model of the road sign.

In this study, the damping parameters of the structure, i.e., the logarithmic decrement of damping (*δ*) and the damping ratio (*ξ*), were determined (see Table 2). The logarithmic decrement of damping is an effective parameter for directly estimating modal damping from the free vibration response of a single mode by fitting an exponential function to the relative maxima of the free decay curve [41]. Based on the experimentally obtained logarithmic decrement of damping (*δ*), the damping ratio (*ξ*) was calculated using the following formula:

(2) $$\xi =\frac{\delta }{2\pi }$$

In the numerical part of this study, the calculation of the dynamic response of the road sign to kinematic excitations caused by mining-induced shocks, the Time History Analysis (THA) method implemented in Abaqus 2022 software was used [42]. This method is based on the direct integration of the equations of motion at each time step. Due to its high accuracy, the THA approach is recommended by European standards [43] for both linear and non-linear dynamic analyses. In the present study, real, recorded mining-induced shocks served as the sources of kinematic excitation affecting the road sign foundation.

In the THA analyses, the Rayleigh model of mass and stiffness proportional damping was used [44]. This model assumes that the damping matrix *C* is a linear combination of the mass matrix *M* and the stiffness matrix *K*:

(3) $$\left[C\right]=\alpha \times \left[M\right]+\beta \times [K]$$

The parameters *α* and *β* in Equation (3) are determined using the natural frequencies and the corresponding damping ratios:

(4) $$\xi_{i}=\frac{1}{2}(\frac{\alpha }{\omega_{i}}+\beta \times \omega_{i})$$

where *ξ_i_* is the damping ratio corresponding to the *i*-th circular frequency *ω_i_*.

### 2.4. The Numerical Model of the Road Sign

**Figure 4:**
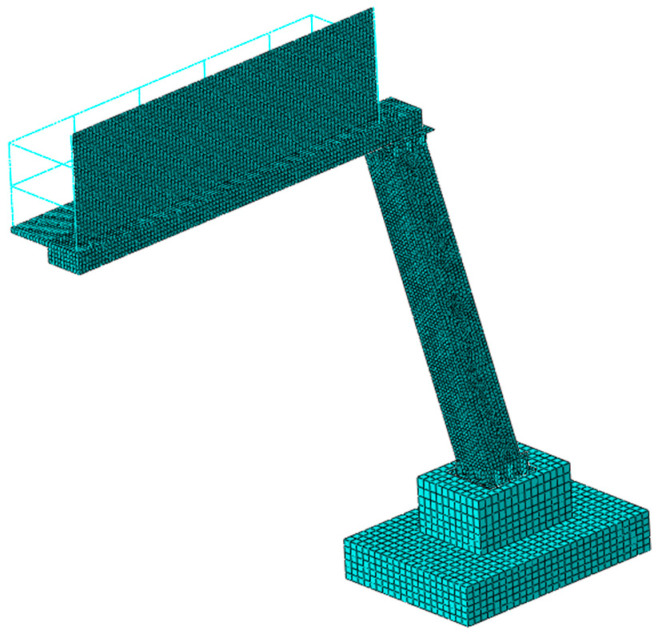
The numerical model of the road sign with the FE mesh.

Using geometric and material data (see Section 2.1), a numerical model of the road sign was developed with Abaqus software. The structural elements of the sign, including the pipe walls forming the inclined support tower and a cantilever arm, were modeled using approximately 12,000 linear quadrilateral elements S4R (4-node 3D shell elements with reduced integration). The non-structural components, such as the railings adjacent to the signboard, were modeled with 470 linear beam elements B31 (linear beams in space). The foundation was discretized with 4340 linear hexahedral elements C3D8R (8-node brick elements with reduced integration). Overall, this model contained about 17,000 elements and 18,000 nodes. Figure 4 illustrates the numerical model of the road sign with the finite element (FE) mesh. This model employs linear-elastic materials with properties listed in Table 1 (see Section 2.1).

**Figure 5:**
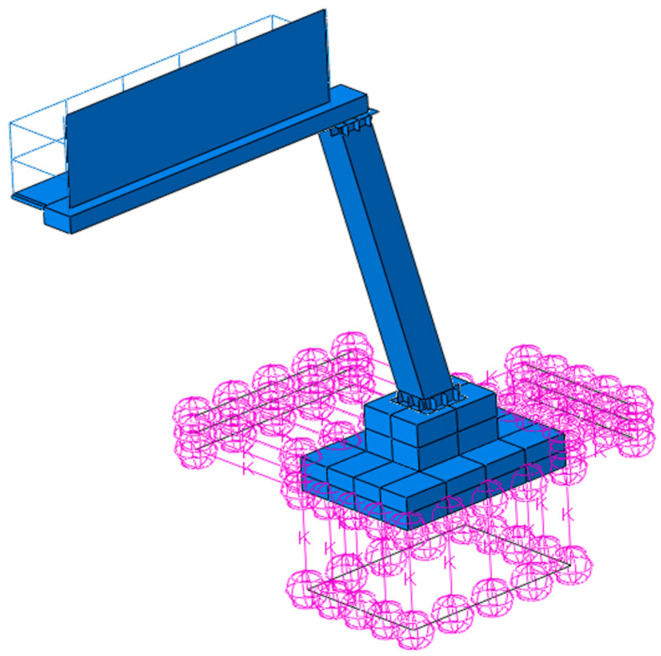
The location of springs reflecting the dynamic soil–structure interaction.

The boundary conditions applied in the initial numerical model reflected the infinite stiff ground (fixed support). However, during the process of tuning, this model was improved by incorporating dynamic soil–structure interaction (DSSI). This enhancement involved the implementation of springs in three directions at the foundation level. The dynamic stiffness of the ground in the vertical ($k_{Z})\;$ and horizontal directions ($k_{X/Y})$ was represented by springs attached to the foundation of the road sign. The number of springs in the vertical direction ($n_{Z})\;$ placed beneath the foundation and attached to its sides in both horizontal directions ($n_{X/Y})$ was 25 and 21, respectively. The location of the springs is shown In Figure 5.

The spring constants, which represent the stiffness of the ground, were calculated based on the Savinov method [45,46]. According to this method, if the contact area is smaller than 50 m^2^, the dynamic stiffnesses $C_{Z}$, $C_{X/Y}$ for a unit area in the vertical and horizontal directions, respectively, can be determined using Equations (5) and (6):

(5) $$C_{Z}=C_{0}\left[1+\frac{2(B+L)}{{∆}_{1}\times (B\times L)}\right]\sqrt{\frac{p}{p_{0}}},$$

(6) $$C_{X/Y}={0.70C}_{z}.$$

where

$C_{0}$—the coefficient for various ground stiffness, recommended in [47] for different types of subsoil [Mpa/m];
*p*—the on-ground static pressure [Mpa];
$p_{0}=0.02\;Mpa$—the reference on-ground static pressure;
$\Delta_{1}=1.0\;m^{-1}$—the dimensional coefficient;
*L*, *B*—the dimensions of the contact area [m].

Based on the coefficients $C_{Z}$, $C_{X/Y}$, the dynamic soil stiffness can be evaluated from Equations (7) and (8) as functions of the contact areas $A_{Z}$ and $A_{X/Y}\;$ in the vertical and horizontal directions, respectively:

(7) $$k_{Z}=C_{Z}\times Az,$$

(8) $$k_{X/Y}=C_{X/Y}\times A_{X/Y}.$$

Considering the number of springs attached to the foundation in the vertical ($n_{z}$) and horizontal ($n_{x/y}$) directions, the stiffness values ($s_{z}$) and ($s_{x/y}$) for the vertical and horizontal springs, respectively, can be calculated from Equations (9) and (10), respectively:

(9) $$s_{Z}=\frac{k_{Z}}{n_{z}},$$

(10) $$s_{X/Y}=\frac{k_{X/Y}}{n_{x/y}}.$$

Based on the soil profile, it was determined that the analyzed sign is found on sandy clay, which, according to the recommendations [47], falls into dynamic ground category I (very low stiffness). For this ground stiffness condition, a coefficient of *C*_0_ = 5 Mpa/m was considered. The static pressure exerted by the sign (11.5T) was calculated as p=0.0205 Mpa. Finally, based on the dimensions of the sign foundation and the number of springs simulating the ground behavior, the vertical and horizontal stiffness constants were calculated according to Equations (5)–(10): *S_z_* = 3 × 10^6^ N/m, *S_x/y_* = 2.6 × 10^6^ N/m.

### 2.5. Characteristics of Mining-Induced Tremors Used for Dynamic Analysis

**Figure 6:**
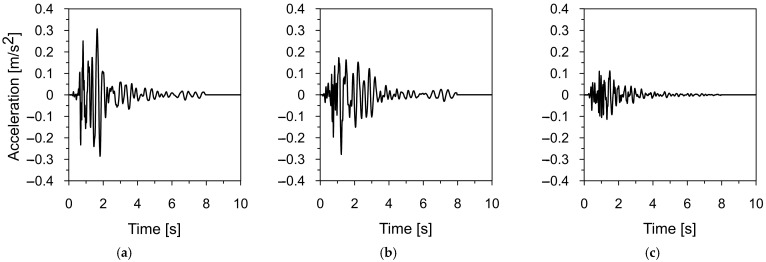
The time-acceleration histories of the Szombierki tremor in (**a**) horizontal direction X, (**b**) horizontal direction Y, (**c**) vertical direction Z.

**Figure 7:**
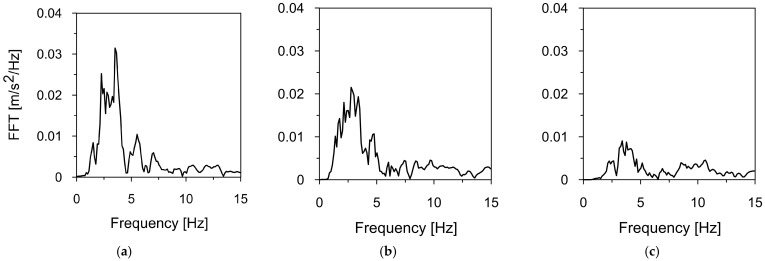
The frequency spectrum of the Szombierki tremor in (**a**) horizontal direction X, (**b**) horizontal direction Y, (**c**) vertical direction Z.

**Figure 8:**
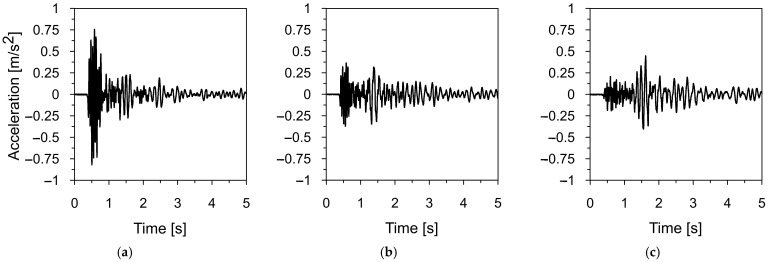
The time-acceleration histories of the Moskorzyn tremor in (**a**) horizontal direction X, (**b**) horizontal direction Y, (**c**) vertical direction Z.

**Figure 9:**
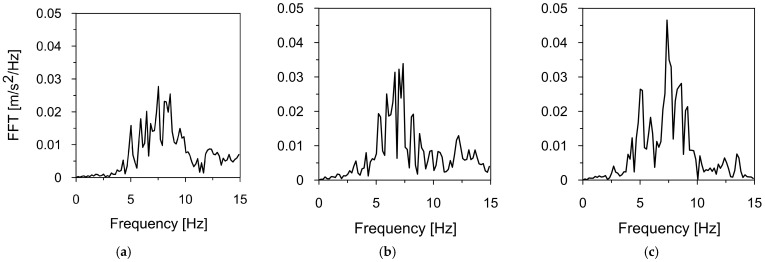
The frequency spectrum of the Moskorzyn tremor in (**a**) horizontal direction X, (**b**) horizontal direction Y, (**c**) vertical direction Z.

Two mining-induced tremors recorded in different mining areas in Poland were selected to assess the dynamic response of the road sign to mining-induced seismicity. The first tremor was registered in Szombierki, located in the Upper Silesian Coal Basin (USCB), while the second occurred in Moskorzyn, within the Legnica-Glogow Copper District (LGCD). Both events fall under the category of high-energy events [48]. The acceleration-time histories and frequency spectra of the Szombierki tremor (registered in three directions) are shown in Figure 6 and Figure 7, respectively. Figure 8 and Figure 9 present the time-acceleration histories and spectral features of the Moskorzyn tremor. A comparative analysis of the key parameters of both tremors is provided in Table 3.

The comparative analysis of the key parameters of both mining-induced tremors revealed significant differences in their characteristics. In terms of energy, the Moskorzyn tremor was five times more powerful than the Szombierki shock. The PGA values also differed between the two recorded tremors. In the horizontal direction, the Moskorzyn shock was nearly double that of the Szombierki. In the vertical direction, the PGA of the Moskorzyn shock was approximately 3.5 times greater than that of the Szombierki event.

It should be emphasized that both tremors fall under the category of strong events [48]. However, predictions for both regions indicate maximum PGA values of 1.8 m/s^2^, which is six times higher than the PGA of the investigated Szombierki tremor and twice as high as the PGA of the analyzed Moskorzyn tremor [12,48].

Another significant difference lies in the dominant frequency ranges. The Szombierki shock exhibited dominant frequencies between 1.6 and 4.8 Hz, with a distinct peak at 3.5 Hz. In contrast, the Moskorzyn event had higher dominant frequencies, ranging from 5 to 10 Hz, with a peak at 7 Hz.

The Szombierki tremor, originating from the Upper Silesian Coal Basin (USCB), and the Moskorzyn tremor, recorded in the Legnica-Głogów Copper District (LGCD), are representative of their respective regions in terms of frequency ranges, tremor duration, and PGA values. Their acceleration-time histories and spectral features align with high-energy mining-induced tremors typically observed in coal and copper mining areas, respectively. Their representativeness provides a strong rationale for selecting these events for in-depth dynamic performance studies, allowing for a comprehensive assessment of the impact of mining-induced seismicity on civil infrastructure elements.

## 3. Results of Numerical Assessment and Field Experiments on Dynamic Properties of the Road Sign

### 3.1. Numerical Estimation of Natural Frequencies and Modes of Vibration

The natural frequencies estimated through the numerical investigation for the assembled FE model of the road sign (see Section 2.4) are summarized in Table 4.

**Figure 10:**
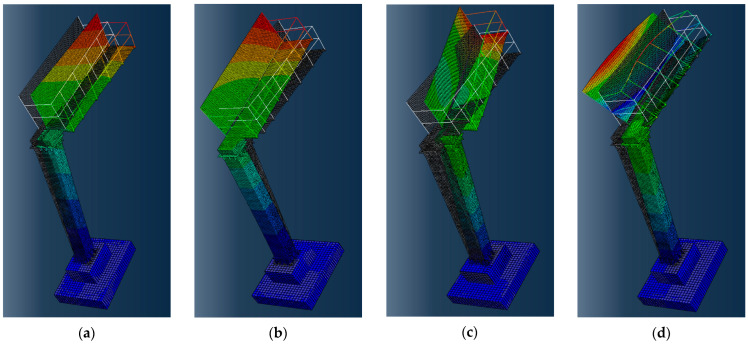
Mode shapes of natural vibrations of the road sign obtained numerically: (**a**) 1^st^ mode, (**b**) 2^nd^ mode, (**c**) 3^rd^ mode, and (**d**) 4^th^ mode (the displacement values are marked with colors: from blue for zero displacement to red for the absolute maximum displacement).

The mode shapes associated with calculated natural frequencies are shown in Figure 10. The first mode shape represents vibrations parallel to the longitudinal axis of the road, whereas the second and third modes are related to vibrations perpendicular to the road axis. Finally, the fourth mode shape is torsional.

It should be emphasized that a simplified model of soil–structure interaction with equivalent springs at the base of the road sign was used in this research. The proposed system of springs, attached to the sides and bottom of the foundation, captured the three-dimensional effects of the local boundary conditions. The stiffness constants of the springs were determined using the Savinov method, which assumes that horizontal soil stiffness is 0.7 times the vertical stiffness (see Section 2.4, Equations (5) and (6)). Based on this assumption, during the initial assembly of the numerical model, the spring constants for sandy clay soil were calculated as follows: $3\times 10^{6}\frac{N}{m}$ in the vertical direction, and $2.6\times 10^{6}\frac{N}{m}$ in the horizontal direction. However, further model validation revealed that the numerically obtained second natural frequency of the road sign, associated with the mode shape in the direction perpendicular to the road axis (see Figure 10b), was more than 10% lower than the experimentally measured value. Thus, this model required tuning. Since the steel structure’s material properties were standard and remained unchanged, the discrepancy suggested that the assumed horizontal stiffness was underestimated. This difference between numerical and experimental frequencies likely arises because the foundation is partially embedded in a subgrade of natural sandy clays and partially in a subbase made of compacted gravel with increased horizontal stiffness. To align numerical results with experimental data, the horizontal stiffness constants were slightly increased to match the vertical stiffness value of $3\times 10^{6}\frac{N}{m}$. The model validation also highlights the strong influence of soil stiffness on the dynamic response of the road sign.

### 3.2. Experimental Estimation of Natural Frequencies and Modes of Vibration

**Figure 11:**
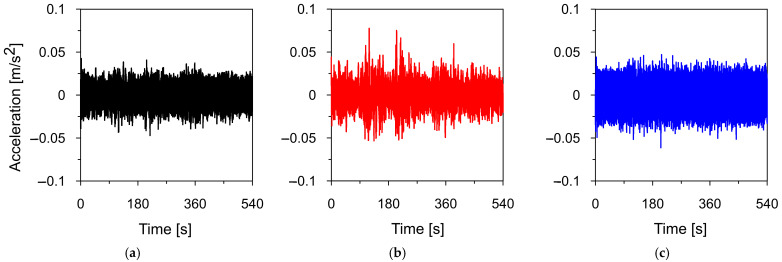
The time-acceleration histories of ambient vibrations caused by vehicle movements in heavy traffic, registered at point P3 (test T1_OMA) in (**a**) X-direction, (**b**) Y-direction, (**c**) Z-direction.

To identify dynamic properties of the road sign in the experimental way, time-acceleration histories resulting from vehicle movements in heavy traffic and light traffic (tests: T1_OMA and T2_OMA, respectively) were registered at three control points P1, P2, and P3 in three directions. The exemplary time-acceleration histories recorded at point P3 during heavy traffic (T1_OMA) are shown in Figure 11.

**Figure 12:**
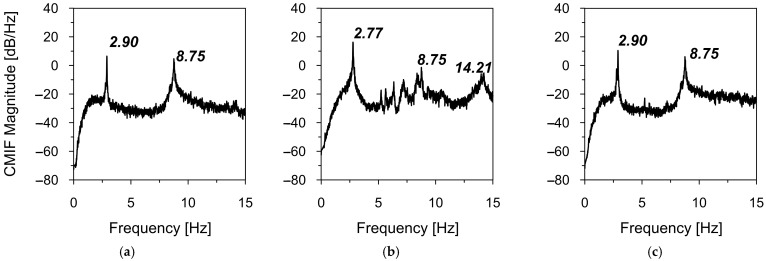
The natural frequency assessment from ambient vibrations caused by vehicle movements in heavy traffic (T1_OMA) with the CMIF in (**a**) X-direction, (**b**) Y-direction, and (**c**) Z-direction.

**Figure 13:**
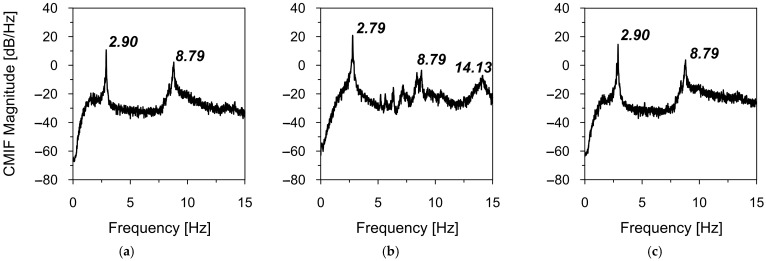
The natural frequency assessment from ambient vibrations caused by vehicle movements in light traffic (T2_OMA) with the CMIF in (**a**) X-direction, (**b**) Y-direction, and (**c**) Z-direction.

Firstly, the natural frequencies of the road sign were assessed based on the complex mode indicator functions (CMIF) (see Section 2.3). The results of the natural frequencies assessment from ambient vibrations caused by vehicle movements in heavy (T1_OMA) and light traffic (T2_OMA) are shown in Figure 12 and Figure 13, respectively.

The local maxima of the calculated CMIFs revealed the first four natural frequencies through their CMIF functions. Table 5 summarizes the natural frequencies identified in both T1_OMA and T2_OMA experimental tests. Strong consistency among the natural frequency values obtained from both experiments is evident, with differences not exceeding 0.5%. These slight discrepancies may be attributed to the accuracy of the measurement set-up (see Section 2.2).

**Figure 14:**
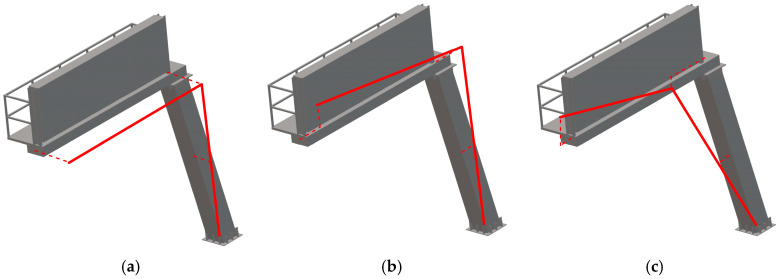
The basic mode shapes of the road sign obtained with the TDD method: (**a**) first mode, (**b**) second mode, (**c**) third mode.

Secondly, modes of natural vibrations were calculated based on the TDD method (see Section 2.3). The basic three-mode shapes obtained from ambient vibrations caused by vehicle movements in heavy traffic (T1_OMA) are shown in Figure 14. It should be emphasized that sensors were placed at only three control points (see Section 2.2), a configuration that allowed for the precise determination of the first three mode shapes.

### 3.3. Validation of the Experimentally Obtained Natural Frequencies and Modes of Vibration

**Figure 15:**
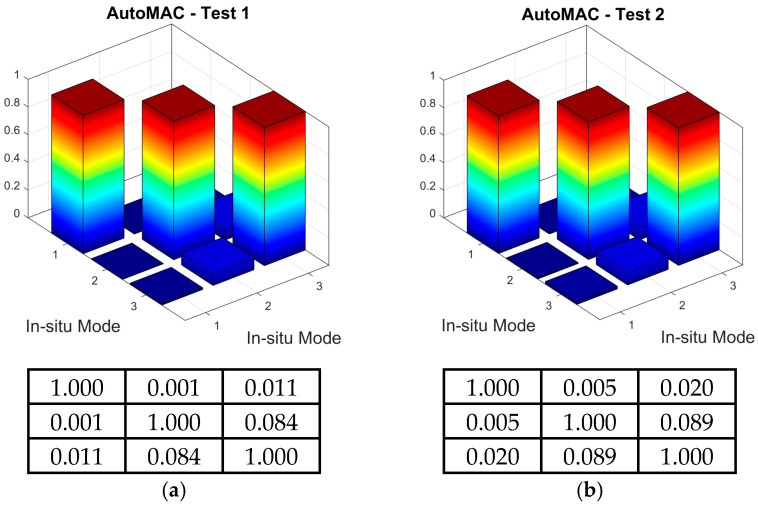
Three-dimensional and matrix presentation of AutoMAC values, serving for positive verification of the experimental modal models obtained from the test: (**a**) T1_OMA, (**b**) T2_OMA.

Once the local maxima of the CMIFs were recognized as the successive natural frequencies and the corresponding modes of vibration were assigned to each frequency, the experimentally obtained dynamic properties were validated using the AutoMAC values. These values were calculated as the self-correlation of the experimental mode shape vectors (see Section 2.3). The AutoMAC values are illustrated in Figure 15 for both the T1_OMA and T2_OMA experimental modal models. It can be observed that all diagonal AutoMAC values equal one, whereas all off-diagonal values are far below 0.2. Therefore, any correlation between the mode shape vectors can be excluded.

**Figure 16:**
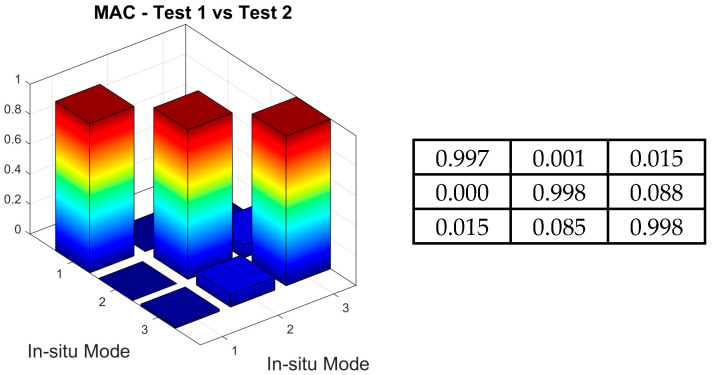
Three-dimensional and matrix presentation of MAC values for the correlation assessment between the T1_OMA and T2_OMA experimental modal models.

Secondly, the MAC tool defined by Equation (1), was used to assess the similarities between the experimental shapes obtained from the T1_OMA and T2_OMA tests. The three-dimensional and matrix representations of the MAC values are shown in Figure 16. Again, all calculated MAC values met the criteria, with values greater than 0.8 along the matrix diagonals and less than 0.2 off the diagonals.

### 3.4. The Numerical Modal Model Validation

The first step of the numerical modal model validation involved comparing the frequencies obtained numerically with those derived from the experimental test T1_OMA. The similarity of the natural frequencies was assessed by analyzing the errors between the numerical and experimental results. The results of the comparison are presented in Table 6. It is clearly visible that strong agreement was found between the numerical and experimental results. The maximal error value did not exceed 6.2%, whereas the mean error value reached 3.7%. The agreement between numerical and experimental results can be considered reasonable, as the differences remain below 7% [49,50]. Similar studies investigating the dynamic characteristics of structures through both experimental and numerical approaches report comparable levels of consistency between measured and computed eigenvalues [18,23]. For example, in the cases of light poles on elevated highway bridges [23] and aluminum highway sign trusses [18], the maximum discrepancies between experimentally identified and numerically obtained fundamental natural frequencies were 1.2% and 4.8%, respectively, with differences of approximately 8–9% in the second and third natural frequencies.

**Figure 17:**
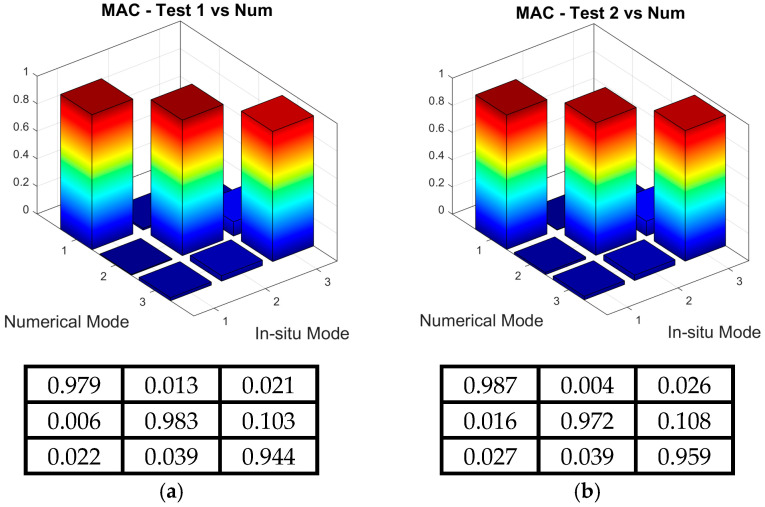
Three-dimensional and matrix presentation of MAC values for the correlation assessment between the numerical and the experimental modal model obtained from (**a**) the T1_OMA test, (**b**) the T2_OMA test.

The second step of the verification process involved analyzing the numerically obtained mode shapes. The MAC criterion (see Section 2.3) was used to assess the similarity between the experimental mode shape vectors from both T1_OMA and T2_OMA tests and the numerical mode shape vectors. Figure 17 presents the MAC values in both three-dimensional and matrix representations, showing that values corresponding to the same modes are close to one, while those associated with different modes are nearly zero. Given the strong agreement between the numerical and experimental results, no further calibration of the FE model was necessary.

### 3.5. Experimental Evaluation of Damping Properties

The estimation of logarithmic damping decrements and critical damping fractions corresponding to the first and second vibration modes was based on discrete experimental data acquired during two series of impacts with the modal hammer (see Section 2.2), each series consisting of five strikes. Since the first mode of vibration was a lateral mode parallel to the roadway axis, the first series of impacts with the modal hammer was conducted in the direction parallel to the roadway axis (test T3_HAM). The second mode of vibration was a lateral mode perpendicular to the roadway axis, so the second series of impacts with the modal hammer was performed in the direction perpendicular to the roadway axis (test T4_HAM).

**Figure 18:**
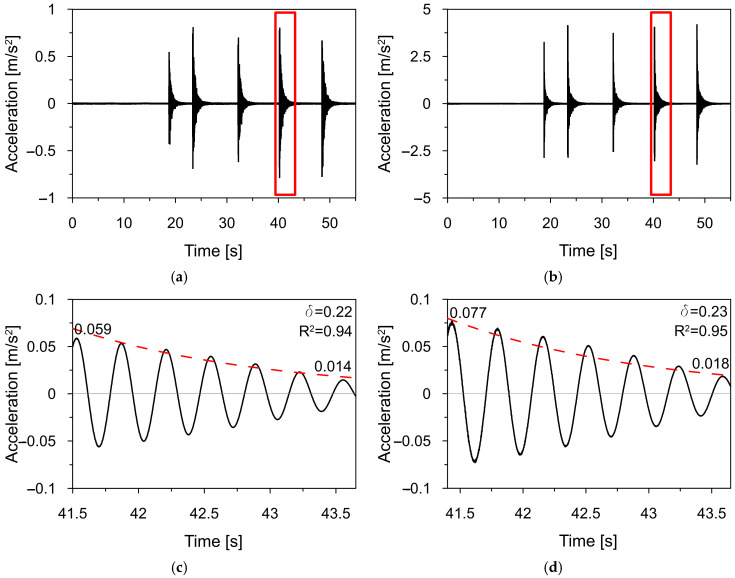
The time-acceleration history recorded during test T3_HAM at point P2 in the direction X (**a**) and Y (**b**), with free decay plots corresponding to the first (**c**) and the second (**d**) mode shape.

The exemplary time-acceleration histories recorded at point P2 in X and Y-directions during the series of five hammer impacts in the test T3_HAM are shown in Figure 18a,b, respectively. To determine the damping properties for the first and second vibration modes, the recorded time-acceleration histories were filtered using a third-order digital Butterworth filter with a bandwidth of 0.02 Hz, centered around the estimated natural frequencies. Based on the least squares regression method, free decay plots (Figure 18c,d) were estimated for all measurement points.

All free decay plots were used to evaluate the values of logarithmic decrements and damping ratios for all strikes in both tests (Table 7). Finally, the average values of the logarithmic decrements were estimated as $\delta_{1}=0.238$ and $\delta_{2}=0.233$ for the first and second natural modes, respectively. The corresponding damping ratios were $\xi_{1}=3.79\%$ and $\xi_{2}=3.71\%$.

Determining the damping parameters of a structure is one of the most challenging aspects of modal analysis due to potential errors from measurement inaccuracies and signal analysis. To validate their findings, the authors compared their results with those from similar studies. In [51], a large box-truss variable message sign structure was analyzed. The average measured in-plane frequency was 1.71 Hz, while the damping ratio reached up to 2.77%. The authors of [52] demonstrated that the cantilevered traffic signal structures exhibited damping values ranging from 2.4% to 6.0%. Similar observations were presented in [53], where the authors investigated a lightweight traffic signal structure. Their research indicated that the addition of supplementary mass to the cantilever section resulted in a significant increase in damping, reaching up to 8%. These comparisons confirm that the obtained damping values are reasonable, supporting the reliability of further calculations.

**Figure 19:**
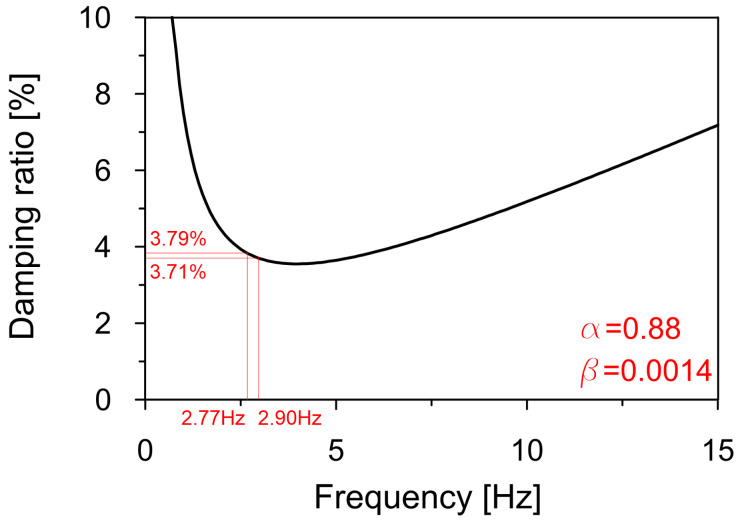
The applied Rayleigh damping model with coefficients α=0.88 and β=0.0014.

Based on the average damping ratios evaluated for the first and second natural frequencies, the Rayleigh damping coefficients (see Section 2.3) were determined as α=0.88 and β=0.0014 (Figure 19).

## 4. Results and Discussion on the Dynamic Performance of the Steel Road Sign with Multi-Material Electronic Signboard Under Mining-Induced Seismicity

### 4.1. Dynamic Response of the Road Sign to the Shocks from Different Mining Activity Regions

The primary objective of this study is to examine how variations in the dominant frequency range affect the dynamic behavior of the sign. To achieve this, its dynamic performance was assessed under mining-induced events from different regions, namely, the Szombierki and Moskorzyn tremors (see Section 2.5). These shocks differ in terms of PGA and dominant frequency range: (1) the Szombierki tremor has a PGA with amplitudes twice as low as those of the Moskorzyn tremor, and (2) the Szombierki tremor has a significantly lower dominant frequency range (1.6–4.8 Hz) compared to the Moskorzyn tremor (5–10 Hz).

**Figure 20:**
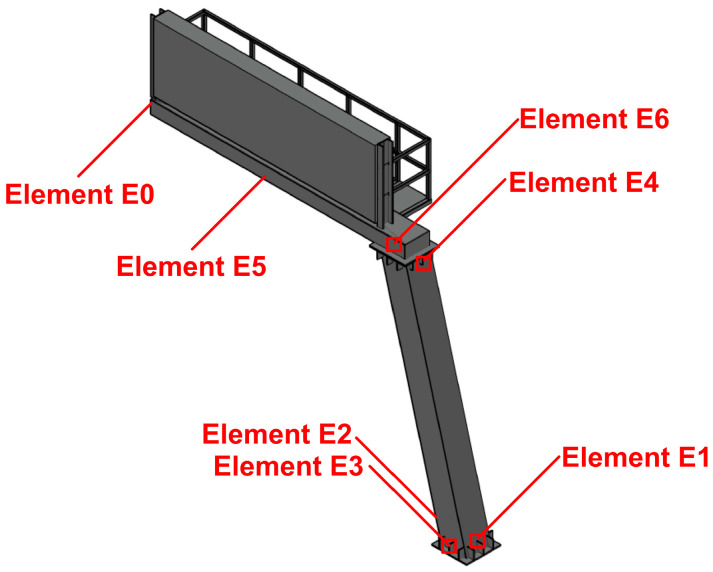
The location of the elements selected for the dynamic analysis.

The dynamic responses of the road sign to the tremors were calculated with the Hilber–Hughes–Taylor time integration algorithm provided in the ABAQUS software for a direct step-by-step solution [42]. The dynamic response levels were assessed in terms of von Mises stresses obtained at six representative elements shown in Figure 20.

**Figure 21:**
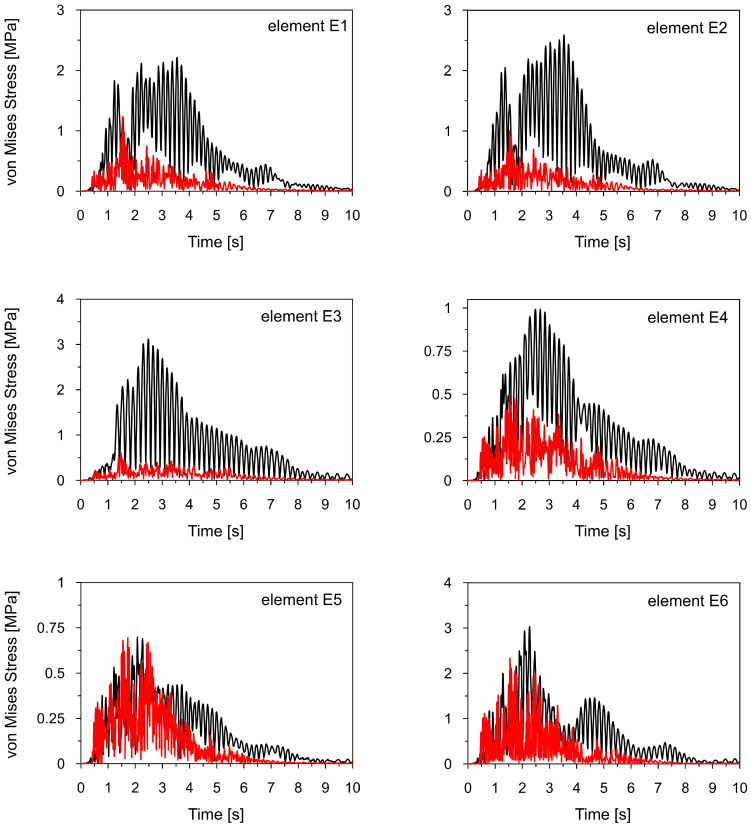
The comparison of the von Mises stresses calculated for the Szombierki (black line) and Moskorzyn (red line) tremors at selected points (see Figure 20).

**Figure 22:**
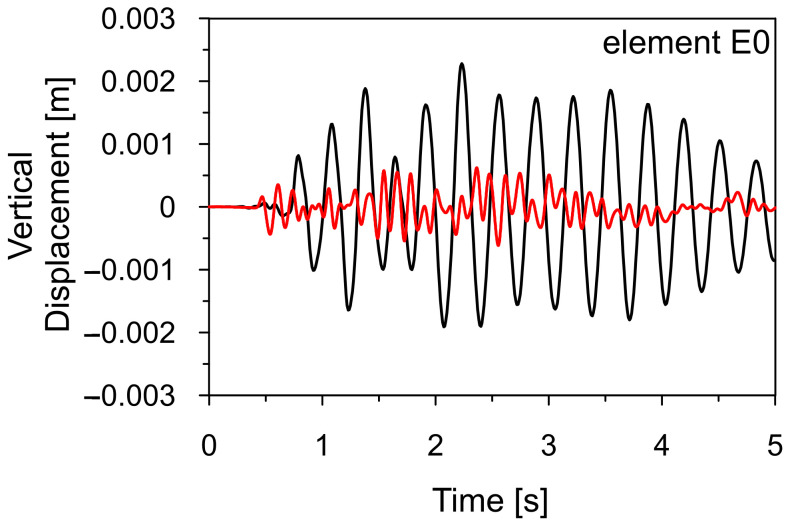
The comparison of the vertical displacement calculated for the Szombierki (black line) and Moskorzyn (red line) tremors at the end of the sign arm E0 (see Figure 20).

Figure 21 compares the time histories of von Mises stresses calculated at elements E1-E6 for the Szombierki and Moskorzyn shocks. Additionally, the comparison between vertical displacements at the end of the sign arm (E0) is presented in Figure 22. The maximum von Mises stress levels obtained during both shocks for all analyzed elements are summarized in Table 8.

**Figure 23:**
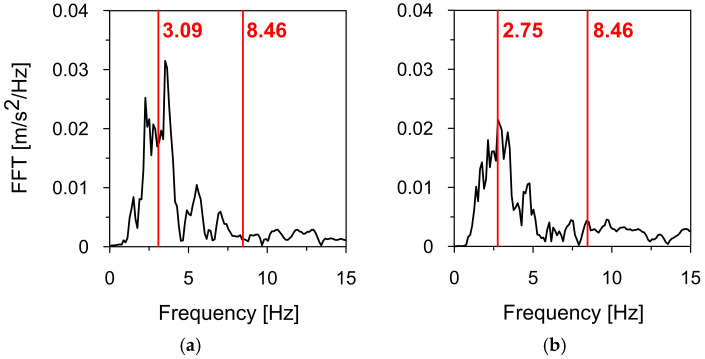
The numerically obtained natural frequencies of the road sign (red lines) vs. the Szombierki tremor’s frequency spectra in (**a**) the direction X, and (**b**) the direction Y.

**Figure 24:**
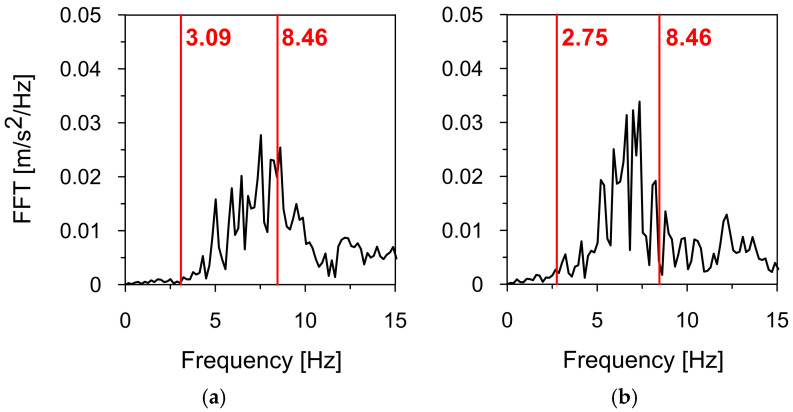
The numerically obtained natural frequencies of the road sign (red lines) vs. the Moskorzyn tremor’s frequency spectra in (**a**) the direction X, and (**b**) the direction Y.

Figure 21 and Figure 22, as well as Table 8, clearly show that the dynamic response level of the road sign is up to five times higher for the Szombierki shock than for the Moskorzyn tremor, despite the Moskorzyn PGA being more than twice as large as the Szombierki PGA. This indicates that the dynamic performance of the sign depends significantly more on the dominant frequency range of mining tremors characteristic of each region than on amplitude levels. These results are illustrated in Figure 23 and Figure 24.

Figure 23 illustrates the numerically obtained natural frequencies of the sign (red lines) against the background of the Szombierki tremor’s frequency spectra in the X and Y-directions. The natural frequency of 3.09 Hz, corresponding to the basic lateral mode shape in the X-direction (perpendicular to the road axis), falls within the dominant frequency range of the Szombierki tremor in the same direction (see Figure 23a).

A similar observation applies to the natural frequency of 2.75 Hz, associated with the basic lateral mode shape in the Y-direction (parallel to the road axis), which is positioned in the middle of the tremor’s dominant frequency range in the Y-direction (see Figure 23b). In contrast, the third natural frequency of 8.46 Hz, corresponding to the lateral mode shape in the X-direction, does not resonate with the frequency spectra in either direction.

A completely different situation occurs when the Moskorzyn tremor affects the sign. Neither the basic frequency of 3.09 Hz in the X-direction (see Figure 24a) nor the basic frequency of 2.75 Hz in the Y-direction (see Figure 24b) falls within the corresponding dominant frequency spectra of this tremor. Only the third vibration mode, at 8.26 Hz, can be amplified by the Moskorzyn tremor. However, exciting higher modes require greater energy, and vibrations associated with these modes typically result in significantly smaller displacements and stresses.

### 4.2. Prediction of the Potential Plastic Behavior of the Multi-Material Electronic Signboard Under Mining-Induced Tremors with the Anticipated Maximum PGA

The objective of this study (see Section 1) was a preliminary assessment of whether typical prefabricated road signs, particularly those with multi-material electronic signboards, constitute a good and safe material solution in mining activity areas where mining-induced seismic events dynamically impact supporting road infrastructure.

This assessment was carried out based on calculations of the dynamic response of the analyzed road sign to a strong mining-induced seismic event. For the calculations, the Szombierki mining-induced seismic event was used as the kinematic excitation; however, its amplitudes were scaled up so that the PGA in the direction of wave propagation reached the maximum value predicted and recorded in the USBC mining area, which is 1.8 m/s^2^ [12].

**Figure 25:**
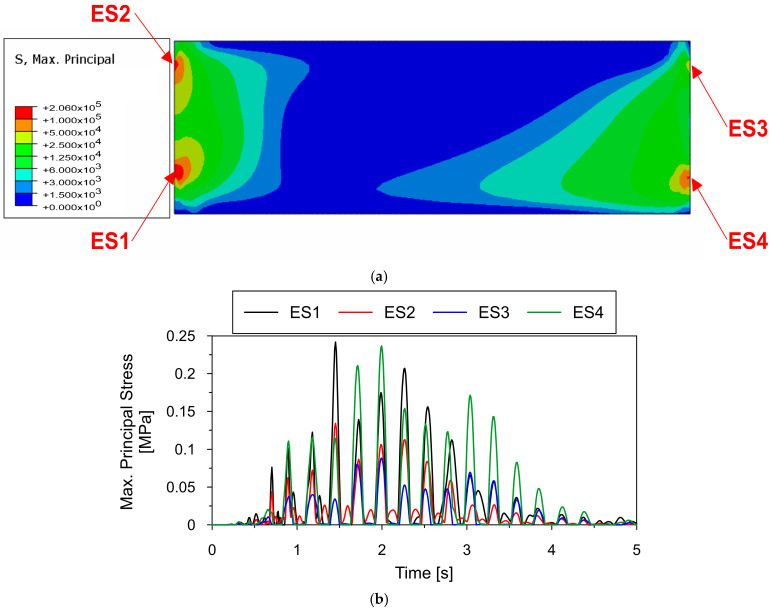
(**a**) The distribution of the maximum principal stress occurring in the multi-material signboard at time t = 1.45 s of the shock, (**b**) the time histories of the maximal principal stresses occurring at points where the signboard is attached to the whole supporting structure.

The distribution of the maximum principal stresses occurring in the homogenized multi-material signboard at time t = 1.45 s of the tremor when its amplitudes reach their maximum values are presented in Figure 25a. In turn, Figure 25b shows the time histories of the maximum principal stresses occurring during the tremor at points where the signboard is attached to the entire supporting structure.

As can be observed from Figure 25, the maximum principal stresses were far below the plasticity limit of the multi-material, which was 50 MPa. None of the points underwent plastic deformation, even in the zones directly adjacent to the connection between the signboard and the supporting structure, where significant stress concentrations occurred. This means that such standard material solutions for signboards can be successfully used in areas of mining-induced seismic activity.

## 5. Conclusions

In this study, the dynamic performance of a road sign subjected to mining-induced seismic tremors recorded in different mining activity regions was examined. The selected events varied significantly in terms of peak ground acceleration and dominant frequency range. This research also included experimental modal analysis to measure the dynamic characteristics of the sign under real conditions and compare the results with numerical simulations using finite element analysis (FEA). The FE model calibration process involved adjusting the boundary conditions to minimize discrepancies between the experimental and computational results. Based on the analyses performed, the following conclusions can be drawn:

- The comparison between the numerically and experimentally obtained natural frequencies showed good agreement. Additionally, the numerical modal analysis, validated through MAC methods, confirmed a close match between the numerical and experimental mode shapes, indicating proper calibration of the FE model;
- The damping properties of the road sign, measured through hammer tests, showed consistent results with similar studies, confirming the reliability of the damping estimation;
- A strong correlation between the numerical simulations and experimental results validated the FE model, enabling further assessment of dynamic performance under mining-induced shocks and ensuring the accuracy of vibration predictions;
- The Moskorzyn tremor had twice the peak ground acceleration (PGA) of the Szombierki tremor; however, it caused five-times lower response stress and displacements. The Szombierki tremor, with a frequency range of 1.6–4.8 Hz, which is closer to the natural frequencies of the sign, led to a significantly higher response level than the Moskorzyn tremor, which had a frequency range of 5–10 Hz. This indicates that frequency matching had a greater impact on the road sign’s response than the PGA;
- The stress levels resulting from both tremors remain well below the permissible limits for the steel structure. In the case of the Szombierki tremor, with a PGA of 0.35 m/s^2^, the maximum von Mises stress observed in the steel structure of the sign reached 5 MPa, while the displacement at the end of the arm was 2 mm. However, the predicted maximum PGA values in this region reach 1.8 m/s^2^—approximately six times higher than the analyzed tremor. Consequently, stresses may increase sixfold, reaching around 30 MPa, with displacements of approximately 1.2 cm. At this level, the stress reaches about 20% of that induced by the dead load;
- The preliminary assessment of the road sign’s dynamic performance showed that the multi-material electronic signboard remained intact, with no plastic deformation or damage. This suggests that such signboards are suitable for use in mining activity areas.

Although the stress levels from high-energy mining-induced tremors remain below the allowable threshold for steel road sign structures, repeated vibrations can still affect their stability, leading to misalignment or shifting. Prolonged exposure to tremors may weaken the structural integrity of sign supports, increasing their susceptibility to external forces such as strong winds or vehicle impacts. For example, the design of the connections between the structure’s column and the foundation, as well as between the column and the cantilever arm, appears to be a critical factor in the context of the obtained results. The precision in constructing these connections and minimizing imperfections in joining structural elements is particularly crucial for signs in mining areas.

Moreover, stress distribution analysis indicates that the highest risk of damage occurs around the column-to-cantilever arm connection and the base of the structure. Elements in these zones—such as anchors, bolts, or welds—are subjected to multiple loading cycles caused by vehicle traffic, wind, and mining-induced shocks. Over time, this repeated loading can lead to material fatigue or cumulative damage, potentially resulting in serious structural failure. These considerations are critical in the design process, particularly regarding durability analysis and the structure’s expected lifespan.

In summary, the results of this study highlight that road sign structures with similar designs may exhibit significantly different dynamic properties under mining tremors due to the unique characteristics of each mining zone. Therefore, before installation, it is crucial to conduct a thorough analysis of the area’s dynamic properties—such as peak ground acceleration and dominant frequency range—along with a predictive modal analysis of the road sign. This ensures an accurate assessment and proper design of the supporting road infrastructure.

## Figures and Tables

**Table 1:** The basic material data for the individual components of the road sign structure.

Structural Element	Material Type	Density [kg/m^3^]	Young Modulus [GPa]	Poisson Ratio [-]	Yield Stress [MPa]
Foundation block	Concrete C20/25	2500	25	0.15	8
Load-bearing structure	Steel St3SX	7850	190	0.3	235
Electronic signboard	Multi-material	260	25	0.3	50

**Table 2:** Description of the experimental tests along with mathematical tools used in analyses.

Test Label	Test Purpose	Description	Mathematical Tools Used in Analyses
T1_OMA	Natural frequencies and mode shapes identification	Ambient vibrations caused by vehicle movements in heavy traffic, lasting for 10 min	Stochastic Subspace Identification (SSI) Complex Mode Indicator Function (CMIF) Time Domain Decomposition (TDD) Modal Assurance Criterion (MAC)
T2_OMA	Natural frequencies and mode shapes identification	Ambient vibrations caused by vehicle movements in light traffic, lasting for 10 min	
T3_HAM	Damping properties evaluation	Modal hammer impacts parallel to the road axis (Y-direction)	Logarithmic decrement of damping (δ) Damping ratio (ξ)
T4_HAM	Damping properties evaluation	Modal hammer impacts perpendicular to the road axis (X-direction)	

**Table 3:** Seismic-induced Shock’s Parameters.

Parameter	The Szombierki Shock	The Moskorzyn Shock
Location	Upper Silesian Coal Basin	Legnica-Glogow Copper District
Duration	6 s	5 s
Strong intensity phase	3 s	2 s
Shock energy	1 × 10^7^ J	5 × 10^7^ J
PGA (WE)	0.35 m/s^2^	0.8 m/s^2^
PGA (NS)	0.28 m/s^2^	0.37 m/s^2^
PGA (vertical)	0.12 m/s^2^	0.45 m/s^2^
Dominant frequency range	1.6–4.8 Hz (all directions) with a peak at 3.5 Hz	5–10 Hz (all directions) with a peak at 7 Hz

**Table 4:** Natural frequencies (Hz) estimated through the numerical investigation.

Natural Frequency (Hz) Estimated Numerically for:			
1st Mode	2nd Mode	3rd Mode	4th Mode
2.75	3.09	8.46	13.50

**Table 5:** Comparison of the natural frequencies (Hz) of the road sign estimated through the experimental tests T1_OMA and T2_OMA.

Experimental Test	Natural Frequency (Hz) Related to:			
	1st Mode	2nd Mode	3rd Mode	4th Mode
T1_OMA	2.77	2.90	8.75	14.21
T2_OMA	2.79	2.90	8.78	14.13

**Table 6:** Natural frequencies (Hz) of the road sign and the percentage error (%) of the numerical frequencies in relation to the experimental results.

Mode No.	Numerical Frequency [Hz]	Experimental Frequency [Hz]	Error [%]
1	2.75	2.77	0.7
2	3.08	2.90	6.2
2	8.46	8.75	3.3
4	13.50	14.21	4.9

**Table 7:** Values of logarithmic decrements and damping ratios obtained for T3_HAM and T3_HAM tests for the first and second-mode shapes.

Test	Strike No.	Damping Properties for the First Mode Shape		Damping Properties for the Second Mode Shape	
		$\mathrm{Logarithmic}\;\mathrm{Decrement}\;\delta_{1}$ [-]	Damping $\mathrm{Ratio}\;\xi_{1}$ [-]	$\mathrm{Logarithmic}\;\mathrm{Decrement}\;\delta_{2}$ [-]	Damping $\mathrm{Ratio}\;\xi_{2}$ [-]
T3_HAM	1	0.254	0.0405	0.272	0.0432
	2	0.223	0.0355	0.261	0.0416
	3	0.232	0.0369	0.236	0.0376
	4	0.230	0.0366	0.224	0.0357
	5	0.243	0.0387	0.221	0.0351
T4_HAM	1	0.271	0.0432	0.230	0.0367
	2	0.243	0.0387	0.225	0.0358
	3	0.218	0.0347	0.195	0.0311
	4	0.251	0.0400	0.231	0.0368
	5	0.217	0.0346	0.236	0.0376
Average value		0.238	0.0379	0.233	0.0371

**Table 8:** The comparison of the maximum von Mises stresses obtained for the Szombierki and Moskorzyn shocks at the selected elements E1–E6 of the road sign.

Element	Maximal Von Mises Stress [MPa]		Ratio
	Moskorzyn	Szombierki	Szombierki/ Moskorzyn
E1	1.24	2.21	1.79
E2	1.00	2.59	2.58
E3	0.61	3.12	5.14
E4	0.49	0.99	2.01
E5	0.69	0.70	1.01
E6	2.33	3.03	1.30

## Data Availability

The original contributions presented in this study are included in the article. Further inquiries can be directed to the corresponding author.
